# Letter to the Editor regarding Meta-analysis of the efficacy of gabapentin: a response

**DOI:** 10.1016/j.xagr.2022.100058

**Published:** 2022-05-05

**Authors:** Ahmed Taher Masoud, Greg Marchand

**Affiliations:** Faculty of Medicine, Fayoum University, Fayoum, Egypt; Marchand Institute for Minimally Invasive Surgery, 10238 E. Hampton, Ste. 212, Mesa, AZ, 85209

Reply to the Letter:

We received the letter to the editor^1^ sent by Vincent et al regarding our systematic review and meta-analysis of the efficacy of gabapentin in chronic female pelvic pain without another diagnosis^2^. We appreciate the opportunity to respond to this letter. In addition, Vincent et al have sent other letters to other journals evaluating articles supporting gabapentin usage. Concerning their other letters, the main point of their response seemed to be to draw attention to the side effect profile of gabapentin. This is peculiar as our meta-analysis specifically focused on the efficacy of gabapentin and not its side effects.

First, the authors claimed that we double-counted patients because we included the study by Seretny et al,^3^ where all patients in the study by Seretny et al^3^ were also included in the study by Lewis et al.^4^ I am uncertain of how meta-analyses are performed at Dr Vincent's institution; however, at our institute, we follow strict adherence to the Preferred Reporting Items for Systematic Reviews and Meta-Analyses (PRISMA) guidelines.^5^ It is appropriate to include both of these studies in the qualitative synthesis; however, it would be misleading and academically irresponsible to not include them, as they both met our inclusion criteria. The PRISMA guidelines are quite straightforward in this case, and care must be taken (and was taken) by our researchers to be sure no double counting occurred. We made sure to avoid comparing both articles under the same meta-analysis. There is not a single result (and thus not a single figure) in which these 2 articles are pooled together, and therefore, there is no double counting.

**Figure:**
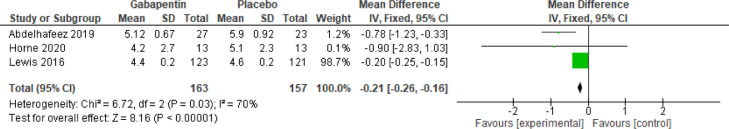
Forest plot of pain with both scores combined *CI*, confidence interval; *IV*, inverse variance; *SD*, standard deviation.^7^, ^8^

Masoud. Meta-analysis of the efficacy of gabapentin. Am J Obstet Gynecol Glob Rep 2022.

Second, regarding the analysis of the visual analog scale and numeric rating scale scores, the Figure shows what would have resulted if both were analyzed under the same outcome.

The Figure shows marked heterogeneity (*I^2^*=70%) among studies, destroying the reliability of the evidence provided here. According to the *Cochrane Handbook for Systematic Reviews of Interventions*,^6^ there are many ways to solve the heterogeneity among studies, and the most common methods used are the leave-one-out method and subgroup analysis. After a careful review of the data extracted from the studies, we found that the different pain scales were the main cause, and therefore, we performed the analyses for each pain score separately to produce a meaningful analysis. Forcibly combining the scales was not appropriate and would lead to a conclusion that not only could be misleading but also could be wrong. We are committed to reporting accurate results honestly, and this was (and still is) the best way to present the results in our opinion.

Third, Vincent et al mentioned that our figures could be misleading because the “end-of-study” number of participants was not equal to the number listed in our table describing the baseline characteristics of the study. To anyone reading any of our studies, we feel that it is obvious that not every study maintains a perfect follow-up with all study participants.

Moreover, our data and percentages were correct and were correctly weighted and entered into our final synthesis. The fact that the final number did not match the initial number of patients is a fact in most major trials. A percentage of “lost to follow-up” is certainly the rule in clinical trials, not the exception.

Lastly, concerning their claims of the danger of leaving out the side effect profile, we sought every means and attempted to design a reliable analysis. The data reported in the included studies do not allow a meta-analysis on safety profiles to be performed. As Vincent et al have given their opinion, we will give ours; our institution has safely used gabapentin to treat hundreds of people suffering from chronic pelvic pain with no serious adverse effect.
